# In-situ sub-angstrom characterization of laser-lubricant interaction in a thermo-tribological system

**DOI:** 10.1038/s44172-024-00284-3

**Published:** 2024-10-05

**Authors:** Qilong Cheng, Sukumar Rajauria, Erhard Schreck, Robert Smith, Qing Dai, David B. Bogy

**Affiliations:** 1 Computer Mechanics Laboratory, University of California at Berkeley, Berkeley, CA USA; 2 Western Digital Corporation, Recording Sub System Staging and Research, San Jose, CA USA

**Keywords:** Mechanical engineering, Applied physics, Characterization and analytical techniques

## Abstract

Laser-lubricant interaction has been a critical reliability issue in a thermo-tribological system named heat-assisted magnetic recording, one of the next generation hard disk drive solutions to increasing data storage. The lubricant response under laser irradiation and the subsequent lubricant recovery are crucial to the system’s reliability and longevity, however, they cannot be diagnosed locally and timely so far. Here, we propose a thermal scheme to in-situ characterize the mechanical laser-lubricant interaction. The nanometer-thick lubricant has a thermal barrier effect on the near-field thermal transport in the system, according to which the lubricant thickness can be determined. As demonstrations, this paper reports the first quantitative in-situ measurements of the laser-induced lubricant depletion and the subsequent reflow dynamics. The proposed scheme shows a sub-angstrom resolution (~0.2 Å) and a fast response time within seconds, rendering in-situ real-time lubricant diagnosis feasible in the practical hard disk drive products.

## Abstract (web summary)

Heat-Assisted Magnetic Recording hard disk drives offer a solution to increasing data storage. Cheng and colleagues demonstrate a near-field thermal transport-based scheme to in-situ measure the lubricant thickness during the lubricant depletion and reflow dynamics, a process crucial to the reliability and longevity of the system.

## Introduction

Near-field thermal transport between two macroscopic bodies separated by a sub-nanometer air gap is of fundamental significance and has high economic value for various industrial applications such as near-field thermophotovoltaics^1^, nanolithography^2,3^ and hard disk drives (HDDs)^4–6^. Most of the studies exploring the fundamental thermal transport modes including phonons and photons are conducted using high-vacuum atomic force microscopy (AFM) or custom-fabricated nano-devices^7,8^. Here, we focus on a thermo-tribological system, the head-disk interface in heat assisted magnetic recording (HAMR) hard disk drives, where the recording heads fly at a sub-nanometer spacing from the disk with a relative speed of 5–40 m s^−1^ (Fig. S1). HAMR uses a laser diode coupled with a near field transducer (NFT) to locally heat the magnetic layer on the disk up to its Curie temperature ( ~ 400–500 °C), which is even hotter than the head temperature ( ~ 150–250 °C)^4,9^. Therefore, to comprehensively understand the thermal transport between the recording head and the disk, which are separated by a sub-nanometer air gap, along with its implications for the associated thin-film components, is critical to the HAMR hard disk drive’s performance and reliability.

Another key feature for the long-term reliability of HAMR hard disk drives is the perfluoropolyether (PFPE) lubricant ( < 2 nm thick) on the disk. The lubricant improves the high-speed tribological performance by passivating the surface against contamination, minimizing friction, and preventing corrosion^4,10^. To guarantee product longevity, the lubricant must be nonvolatile, and have sufficient mobility to replenish areas where the lubricant has been depleted due to possible high-speed head-disk contact^11^. Moreover, due to the introduction of the laser in HAMR, the hot spot on the disk ( ~ 400–500 °C) is well beyond the lubricant evaporation temperature ( ~ 150–250 °C)^12,13^, so the lubricant undergoes evaporation, decomposition and diffusion under thermal exposure^10^. After evaporation, the lubricant can condense on the head surface and accumulate there as “smear”^10,14^. Therefore, an in-depth understanding of the laser-lubricant interaction under HAMR operating conditions is of great importance to the reliability of the HAMR hard disk drives. On the other hand, the lubricant has a self-healing ability, and this is also an indispensable property for the sake of reliability. Some of the depleted lubricant recovers gradually due to the lubricant reflow, which has mostly been studied using ex-situ methods^15,16^. An in-situ measurement of the lubricant reflow is valuable to the tribological longevity of HAMR hard disk drives.

In this work, we propose a near-field thermal transport based scheme to in-situ measure the lubricant thickness during the lubricant depletion and the lubricant reflow. The effects of the lubricant thickness on the near-field thermal transport across the head-disk interface are first investigated. By use of the effects, we determine the dynamic lubricant thickness under various operations. As demonstrations, this paper reports the first quantitative in-situ measurements of the laser-induced lubricant depletion and the subsequent reflow dynamics. The proposed scheme shows a sub-angstrom resolution ( ~ 0.2 Å) and a fast response time within seconds, rendering in-situ real-time lubricant diagnosis feasible in the practical HDD products.

## Results and discussion

### HAMR Head-Disk Interface

**Fig. 1:**
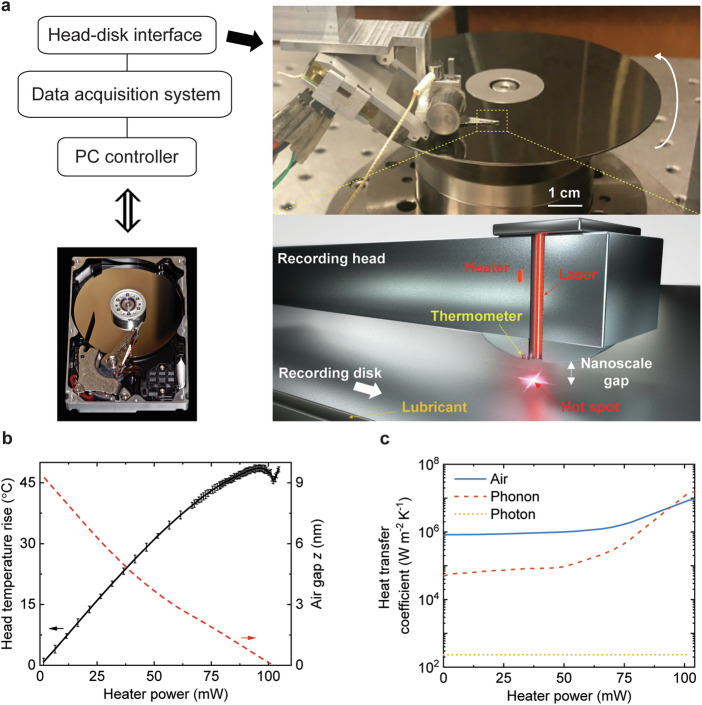
Experimental platform. **a** The component-level heat assisted magnetic recording (HAMR) test stage. A data acquisition system connected to a personal computer (PC) controls components in the head (heater, laser) and acquires the thermometer signal. This setup mirrors a HAMR hard disk drive (HDD) as all operations here are common and feasible in the HAMR HDD. **b** The measured thermometer’s temperature rise *T* and the simulated air gap *z* as a function of the heater power *P* in the laser-off condition. The temperature undergoes three regimes that are dominated by joule heating, cooling, and frictional heating, respectively. The air gap was simulated using a commercially available program named CMLAir. **c** The calculated heat transfer coefficients due to air conduction, phonon heat conduction and radiation carried by photons.

A HAMR recording head and a HAMR disk were used for such a thermal transport study. The experimental setup is a component-level HAMR test stage (Fig. 1a), where the head flies over the disk with a closing air gap from 10 to 15 nm to contact. It is a unique thermo-tribological platform in which the head’s spacing from the disk is precisely controlled with a sub-nanometer resolution using an embedded microscale heater, and the head has an active integrated thermometer of ~1 μm long, which is used to measure the head temperature for various air gaps^17,18^. The heater, also known as thermal fly-height control in the HDD industry, generates a microscale thermal protrusion on the head surface (the bulge in Fig. 1a) by thermal expansion to adjust the air gap between the head surface and the disk surface. Also, the HAMR head has an integrated laser diode that is used to locally heat the disk to a temperature higher than the Curie temperature of the magnetic layer ( ~ 400–500 °C)^5,6^. It is noted that this head has a waveguide but no NFT because the waveguide head provides a larger laser spot size ( ~ 300 nm) than that in the NFT head ( ~ tens of nanometers)^4–6^, and hence works better with the thermometer. At the other side of the air gap, the rotating disk has a FePt-based magnetic layer on a glass substrate that is coated with an amorphous carbon overcoat (protective layer, several nanometer thick) and a molecular layer of PFPE lubricant ( < 2 nm thick)^4^. This experimental setup mirrors a real HAMR HDD, as all operations such as changing heater power, turning on/off laser, and monitoring thermometer signal are common and feasible in the HAMR HDD in-situ.

The head surface temperature was monitored for various air gap values to measure the thermal transport across the head-disk interface while the disk was rotating at a speed of 15 m s^−1^ (5400 RPM) relative to the stationary head, which is six orders of magnitude higher than that in the previous AFM-based studies^7^. To determine the air-gap dependent thermal transport across the head-disk interface in the laser-off condition, we measured the thermometer’s temperature rise (*T*) as the air gap was decreased by increasing the heater power (*P*). Figure 1b shows the temperature rise as a function of the heater power and the simulated air gap *z*^19^. The relationship between the air gap *z* and the heater power was obtained using a commercially available program named CMLAir. As the head surface approaches the disk, the thermometer’s temperature evolution with the heater power can be sub-divided into three regimes (black dots in Fig. 1b). An initial increase in the thermometer temperature is due to joule heat dissipation in the heater, followed by a drop in the thermometer temperature at a narrower gap (*z* < ~1 nm) where the enhanced near-field thermal transport overcomes the joule heat dissipation, and finally a sharper increase in the thermometer temperature is due to frictional heating produced by the high-speed head-disk contact, which occurs beginning at the heater power of 102 mW (contact onset). Meanwhile, the relation between the air gap *z* and the heater power shows nonlinearity when the gap narrows, which is due to air pushback force^20^. With an increasing heater power, the heater protrusion gradually closes the air gap. A smaller air gap induces a larger air bearing pressure and a stronger pushback force from the air bearing to the heater protrusion, lowering the heater efficiency (nm per milliwatt). Therefore, the heater narrows the air gap with an efficiency of 0.1 nm per milliwatt when the gap is ~8 nm, but this efficiency decreases to 0.05 nm per milliwatt for air gaps less than 1 nm.

Figure 1c shows the simulation results of the heat transfer coefficients (HTCs) across the head-disk interface due to air conduction^21,22^, phonon heat conduction^23^ and radiation^24^. Here, the heat transfer related to the air is dominated by the conduction term, not the convection term^21,25^. The simulations in Fig. 1b, c is performed using a thermo-mechanical head-disk interface model, where the details can be found in Ref. ^19^. Figure 1c shows that the radiation carried by photons is negligible compared to the two other mechanisms. Thus, the total head-disk heat transfer coefficient (HTC_head-disk_) can be approximated as^19^,

1 $${{{{\rm{HTC}}}}}_{{{{\rm{head}}}}-{{{\rm{disk}}}}}\approx {{{{\rm{HTC}}}}}_{{{{\rm{air}}}}}+{{{{\rm{HTC}}}}}_{{{{\rm{phonon}}}}}$$

where HTC_air_ and HTC_phonon_ are the heat transfer coefficients for the heat conduction by air and by phonons, respectively. They have comparable values when the heater power is larger than 88 mW (*z* < ~1 nm).

### Thermal Effects of the Lubricant

**Fig. 2:**
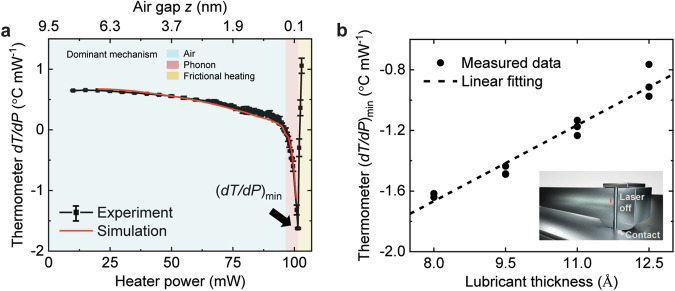
Thermal barrier effect of lubricant. **a** The rate of change of the thermometer temperature rise with the heater power, *dT/dP*, as a function of the heater power and the corresponding air gap size. The *dT/dP* drops gradually, and its minimum (*dT/dP*)_min_ occurs right before contact. **b** The extracted thermometer (*dT/dP*)_min_ and its linear fitting as a function of the lubricant thickness. This calibration result is later used to determine the lubricant thickness in the following experiments of lubricant depletion and lubricant reflow. Inset: a sketch for the (*dT/dP*)_min_, corresponding to the maximum thermal transport.

To better interpret the experimental results of the thermal transport, the rate of change of the thermometer temperature rise with the heater power, *dT/dP*, was extracted and plotted in Fig. 2a, along with the simulation result. The contact onset is at the heater power of 102 mW. Figure 2a shows that the *dT/dP* monotonically decreases with the gap before contact occurs, indicating an increasing heat transfer coefficient between the head and the disk at a smaller air gap.

In Fig. 2a, the *dT/dP* is positive and decreases gradually for the large air gap regime (heater power <96 mW, *z* > ~0.4 nm) where the air conduction generally dominates the head-disk thermal transport as shown in Fig. 1c. A positive *dT/dP* indicates that the heat transfer coefficient from the heater to the thermometer is larger than HTC_head-disk_, so the heater heats up the thermometer. However, with the increasing heater power, the air gap narrows and the HTC_head-disk_ increases rapidly (Fig. 1c), leading to a negative *dT/dP*. Then, at very small air gaps (96 mW <heater power <102 mW, *z* < ~0.4 nm), the *dT/dP* drops steeply and goes below zero as the nonlinear phonon heat conduction begins to dominate cooling of the head thermometer. Moreover, the good agreement between the experimental result and the simulation result of *dT/dP* as shown in Fig. 2a testifies that the phonon heat conduction is dominant at the sub-nanometer sized air gap between the head and the disk.

Here, the effects of the lubricant on the near-field thermal transport at the small head-disk gap (*z* ~ several Å) are investigated. The *dT/dP* was measured as a function of the heater power on disks with the lubricant thickness varying from 8.0 to 12.5 Å, as shown in Fig. S2. As the head approaches the rotating disk, the rate of change of the head temperature versus the heater power, *dT/dP*, decays fast with the head-disk gap size and then increases due to the frictional heating after contact occurs, where the minimum (*dT/*dP)_min_ occurs at the heater power of 101.5 mW. The (*dT/*dP)_min_ for each lubricant thickness was extracted from the red dashed box in Fig. S2, corresponding to the maximum of total thermal transport including air conduction, phonon heat conduction, contact heat conduction and frictional heating. Figure 2b shows the extracted (*dT/*dP)_min_ as a function of the lubricant thickness, where the inset shows a sketch for the (*dT/*dP)_min_. The disk with a thicker lubricant provides a larger (*dT/*dP)_min_ indicating a relatively stronger thermal barrier effect, while the disk with a thinner lubricant shows a smaller measured (*dT/*dP)_min_ and, thus, more cooling.

To further study the thermal effects of the lubricant, Fig. S3a plots the simulated *dT/dP* as a function of the HTC across the head-disk interface using the model in Ref. ^19^. With the decrease of the air gap between the head and the disk, the increasing HTC causes a smaller *dT/dP*. By use of this relation, the measured (*dT/*dP)_min_ in Fig. 2b was converted into maximal total HTC as shown in Fig. S3b. When the lubricant thickness increases by 1.0 Å, the maximal total HTC decreases by 2.76 × 10^6^ W m^−2^ K^−1^), which demonstrates the effect of the lubricant as a thermal barrier on the thermal transport between the head and the disk. It is known that the thermal conductance (*tc*) of the lubricant layer can be expressed as^26^,

2 $$\frac{1}{tc}=\frac{h}{k};k=\frac{{k}_{{{{\rm{bulk}}}}}}{1+\frac{4{\varLambda }_{{{{\rm{bulk}}}}}}{3h}}$$

where *h* is the lubricant thickness, *k* is the effective thermal conductivity of the lubricant considering boundary scattering using Matthiessen’s rule^27^, *k*_bulk_ is the bulk thermal conductivity, and *Λ*_bulk_ is the bulk mean free path of the thermal energy carrier. Equation 2 shows that the lubricant’s thermal conductance (*tc*) decreases with its thickness (*h*), which qualitatively matches the previous experimental findings. This (*dT/*dP)_min_ measurement provides another approach to characterize the near-field thermal transport across the head-disk interface. In the following, we use the near-field thermal transport ((*dT/*dP)_min_) at the small head-disk gap (*z* ~ several Å) to in-situ measure the lubricant depletion and reflow dynamics according to Fig. 2(b).

### Lubricant Depletion under Laser Irradiation

Here, we track the lubricant depletion dynamics by measuring the maximal thermal transport between the head and the disk, namely the (*dT/*dP)_min_, as the lubricant is evaporated under thermal exposure. The head flying above the disk has an integrated laser diode to heat the disk to a temperature higher than the Curie temperature of the magnetic layer ( ~ 400–500 °C) to assist data writing^4^. By making use of the HAMR writing, we calibrated the disk peak temperature at each laser power condition as previously done (Fig. S4)^14^.

**Fig. 3:**
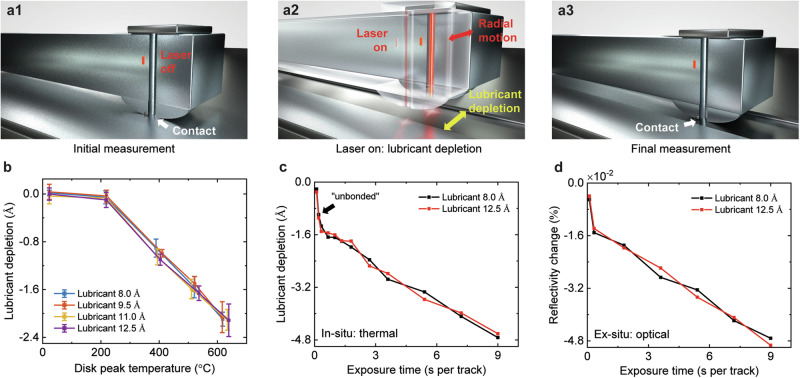
Lubricant depletion under laser irradiation. **a1**–**a3** Experimental scheme: **a1** initial measurement of the original lubricant thickness; **a2** lubricant depletion with laser on; **a3** final measurement of the lubricant thickness after depletion. **b** The lubricant depletion as a function of the disk peak temperature at the laser exposure time of 1.8 s per track. The lubricant depletion occurs at temperatures higher than 220 °C. **c** The lubricant depletion and **d** the reflectivity change as a function of the laser exposure time at the disk peak temperature of 650 °C. Their excellent agreement verifies the capability of the in-situ thermal scheme to track the lubricant depletion. Error bars are <0.2 Å and <0.001%.

Figure 3(a1–a3) illustrates the experimental scheme for characterizing the depletion dynamics of the lubricant as the disk was exposed to high temperature. First, the thermal transport was measured using the *dT/dP* as a function of the air gap on the original disk (Fig. 3(a1)). Then, the disk was heated to a constant temperature using the built-in laser diode, and the lubricant was depleted over a band of width ~10 μm (Fig. 3(a2)). Considering that the laser spot size is ~300 nm, which is smaller than the thermometer’s length ( ~ 1 μm), we moved the head back and forth by ~10 μm in the radial direction to generate a uniform band. Finally, we re-measured the *dT/dP* on the lubricant-depleted band as a function of the air gap (Fig. 3(a3)) to quantify the change in the (*dT/*dP)_min_ to derive the lubricant depletion, according to the calibration curve in Fig. 2b. This in-situ scheme has a resolution ~0.2 Å.

Figure 3b shows the in-situ measured lubricant depletion as a function of the disk peak temperature, where the accumulated laser exposure time was 60 s for the whole band and each track was heated by 1.8 s on average. All of the four lubricant thicknesses show similar results. When the disk peak temperature is lower than 220 °C, there is almost no reduction in the lubricant thickness, which indicates the lubricant’s thermal stability. For temperatures higher than 220 °C, the lubricant depletion is proportional to the disk peak temperature rise. It is noted that the in-situ measured thermal stability of the lubricant bonded on a disk matches the bulk thermogravimetric analysis (TGA) experiments for the PFPE lubricant^28^. At the same laser power, the lubricant depletion can vary with the radius because the HTC_head-disk_ at the outer diameter (OD) with a higher linear velocity is larger than that at the inner diameter (ID), affecting the temperature rise at the disk. However, during data writing in HAMR technology, the disk needs to be heated to Curie temperature by applying laser power, which can be ~18% higher at OD than at ID^29^. Under the same disk temperature conditions (e.g., Curie temperature), the lubricant depletion should be independent of the radius.

We also explore the temporal evaporation dynamics of the lubricant depletion at a constant disk peak temperature of 650 °C. The thermometer *dT/dP* at the close spacing was measured to quantitatively determine the lubricant depletion at different laser exposure times. Figure 3c shows the lubricant depletion as a function of the laser exposure time. Interestingly, the initial 1.5 Å loss happens at the rate 3.4 Å s^−1^, which is much faster than the subsequent lubricant loss at the rate 0.37 Å s^−1^. We attribute this larger rate to the depletion of unbonded lubricant, which can be evaporated faster. To further confirm this behavior, we used an ex-situ optical surface analyzer (OSA, Candela 5100) to characterize the lubricant uniformity on the heated disk. Figure 3d shows the reflectivity change before/after the depletion as a function of the exposure time. The excellent agreement between the in-situ thermal transport based and the ex-situ optically based techniques verifies that the thermometer *dT/dP* measurement at the close spacing can track the lubricant depletion that occurs under the high-temperature HAMR operations. Therefore, this in-situ scheme can serve as a practical application for tracking lubricant depletion in HAMR hard disk drive technology.

### Lubricant Reflow Dynamics

**Fig. 4:**
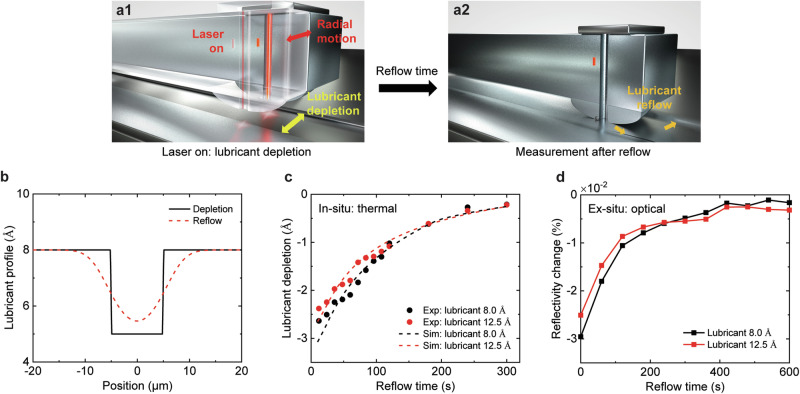
Lubricant reflow dynamics. **a1–a2** Experimental scheme: **a1** lubricant depletion with laser on; **a2** measurement of the lubricant thickness at the center of the band after a specific reflow time. **b** Schematic lubricant profiles of depletion and reflow. The depletion curve was assumed as the initial condition of the reflow process. The reflow curve was obtained by solving Eq. 3. **c** The lubricant depletion and **d** the reflectivity change as a function of the reflow time after the disk was heated for 3.0 s per track at 650 °C. Their excellent agreement demonstrates that the in-situ thermal scheme can be used to diagnose the lubricant’s reflow capability. Error bars are <0.2 Å and <0.001%.

Since the lubricant layer undergoes evaporation, decomposition and diffusion under thermal exposure, its self-healing ability, also known as reflow, is crucial to reliability. Here, the reflow dynamics of the lubricant was quantitatively tracked by measuring its thickness over time after the thermal exposure, using the thermometer *dT/dP* at the close spacing. Figure 4(a1–a2) illustrates the experimental scheme. First, a depletion band was made on the disk using the built-in laser diode in the head (Fig. 4(a1)), which is the same as Fig. 3(a2). The disk was exposed to a high temperature of 650 °C for 3.0 s per track such that the lubricant was depleted by ~3 Å. Then, the laser was switched off for some time to allow the lubricant reflow, and the lubricant thickness at the center of the band was measured as a function of the reflow time. The disk was still rotating during the reflow process, otherwise the head would crash onto the disk. Schematic lubricant profiles for the depletion and the subsequent reflow are plotted in Fig. 4b.

Figure 4(c) shows the measured lubricant thickness using the thermometer *dT/dP* at the close spacing as a function of the reflow time. The black and red dots correspond to the reflow dynamics on the two disks with initial lubricant thicknesses of 8.0 and 12.5 Å, respectively. The governing equation for the reflow dynamics can be derived using continuum theory as^16,30^:

3 $$\frac{\partial h}{\partial t}+\frac{1}{3\mu }\frac{\partial }{\partial x}\left[{h}^{3}\frac{d\varPi (h)}{dh}\frac{\partial h}{\partial x}\right]=0$$

where *h* = *h*(*x*,*t*) is the lubricant thickness, *μ* is the effective viscosity, $\Pi \left(h\right)=A/{h}^{3}$ is the disjoining pressure due to van der Waals interactions between the lubricant and the disk carbon overcoat with *A* being the Hamaker constant. The governing partial differential equation was solved numerically. The dashed lines in Fig. 4c show the simulated reflow using Eq. 3 with the unknown Hamaker constant *A* as a fitting parameter (*A* = 4.5 × 10^−21^ J, close to the value used in Ref. ^31^. It is noted that the time constant for the lubricant reflow is ~120 s for both thicknesses. To independently confirm the reflow dynamics, the lubricant reflow was also measured using the ex-situ OSA. In Fig. 4d, the black and red dots show the reflectivity change with respect to the non-heated condition as a function of the reflow time on the two disks. At *t* = 0 s, the absolute value of the reflectivity change is large due to the in-situ lubricant depletion as the disk is exposed to the high temperature. Subsequently, the laser is turned off, and the reflectivity change gradually goes to zero indicating that the lubricant reflow occurs, with a similar time constant. It is worth noting that the in-situ thermal transport based and the ex-situ optically based techniques show an excellent match for both the lubricant depletion and the reflow dynamics. In addition, this in-situ scheme only takes ~1 s, while the ex-situ OSA takes ~20 s because an annular region needs to be scanned. Hence, the in-situ scheme works more efficiently to capture the reflow dynamics precisely and timely, which can be implemented to diagnose the lubricant’s reflow capability throughout its lifetime, a key property for ensuring the longevity of HAMR technology.

This in-situ scheme for measuring lubricant thickness relies on the (*dT/dP*)_min_ measurement, which has a resolution of 0.04 °C mW^−1^. Accordingly, the lubricant thickness measurement achieves a resolution of ~0.2 Å, as illustrated in Fig. 2b. Three repetition measurements were performed for each data point in this work. Figure 3b shows a standard deviation of 0.08–0.19 Å. Compared with the reflectivity data given by the OSA, this in-situ scheme shows an accuracy of ~90%.

## Conclusions

In summary, we performed a near-field thermal transport study using the HAMR head-disk interface, which is a unique thermo-tribological system for exploring the fundamental thermal transport mechanisms between two macroscopic bodies separated by sub-nanometer air gaps. The thermal transport is dominated by air conduction and phonon heat conduction between the head and the disk, where the relative speed is six orders of magnitude higher than that in the previous AFM-based studies. The good agreement between the *dT/dP* measurement and the simulation provides the evidence that the phonon heat conduction is dominant at the operating sub-nanometer sized air gap between the head and the disk. Furthermore, the effects of the lubricant thickness on the thermal transport between the head and the disk were investigated. The lubricant layer behaves as a thermal barrier in that an increase of 1.0 Å in the lubricant thickness causes the decrease of 2.76 × 10^6^ W m^−2^ K^−1^ in the maximal HTC across the head-disk interface.

By use of the lubricant’s thermal effects, we proposed the in-situ scheme to measure the lubricant thickness, and reported the first quantitative measurements of the sub-angstrom depletion and reflow dynamics of the nanometer-thick PFPE lubricant on the disk when it was exposed to the high-temperature HAMR-type operations. This in-situ scheme has a sub-angstrom resolution ( ~ 0.2 Å) and a faster response time than the ex-situ method (OSA). Since all operations in this work are common and feasible in HAMR hard disk drives, the proposed scheme can be implemented in HAMR products for in-situ real-time lubricant diagnosis without the need for ex-situ tools such as OSA or ellipsometer. We envision that the insights obtained from this study will be important for future technology including HAMR hard disk drives, where two macroscopic objects are at a sub-nanometer spacing.

## Methods

### Setup

The experiments were performed using a component-level hard disk drive test stage. The recording heads and the recording disks with different lubricant thicknesses 8.0–12.5 Å were provided by Western Digital Corporation. In the experiments, the recording head was flying on top of the rotating disk (5400 RPM) with a relative speed of 15 m s^−1^. The elements in the head (heater, laser, thermometer) were controlled by a data acquisition system that consisted of two synchronized Multifunction I/O Devices manufactured by National Instruments: PCI-6115 and USB-6211. They have multiple analog output channels and analog input channels with high sampling rate ( ~ MHz). The heater power was increased until contact occurred with the details in the next section. The laser was energized by a DC voltage source, and the thermometer was kept by a current source at 2.5 mA to sense the cooling effect near contact with a good sensitivity.

### Air-gap control

The air gap between the head and the disk was ~10–15 nm initially due to the air bearing design on the head surface, and then was reduced to zero using the heater element in the head. The heater generated a protrusion on the head surface and thus narrowed the air gap. The heater was energized by a DC voltage controller with an increasing power of 5 mW initially and 0.5 mW near the contact onset. Each heater power was maintained for 0.11 s (10 revolutions of the disk rotation). The time constant of the heater is below microsecond. The heater power was increased to contact onset plus 2 mW, where the contact onset was detected by using an acoustic emission sensor. The relationship between the heater power and the air gap size was obtained using a commercially available program named CMLAir.

### Simulations

The air gap in Fig. 1b was computed using a commercially available program named CMLAir. The simulations of heat transfer coefficients in Fig. 1c and the *dT/dP* in Fig. 2a are performed using a thermo-mechanical head-disk interface model, where the details can be found in Ref. ^19^. The lubricant reflow dynamics in Fig. 4c was simulated by a MATLAB script solving Eq. 3, where the spatial derivative was approximated using the centered finite difference scheme (second order accurate) and the time derivative was discretized using the forward Euler method.

### Reporting summary

Further information on research design is available in the Nature Portfolio Reporting Summary linked to this article.

## Supplementary information

Supplementary Information

Reporting Summary

## Data Availability

The data that support the findings of this study are available from the corresponding author upon reasonable request.
