# Supplementary material for: In-situ sub-angstrom characterization of laser-lubricant interaction in a thermo-tribological system
Source: Commun Eng. 2024 Oct 5;3:138. doi: 10.1038/s44172-024-00284-3 (PMC11457497; doi:10.1038/s44172-024-00284-3)
Supplement: Supplementary file 1 — Supplementary Information [file 44172_2024_284_MOESM1_ESM.pdf]

Supplementary Information for

In-situ sub-angstrom characterization of laser-lubricant interaction in a thermo-tribological system

Qilong Cheng[1,2]*, Sukumar Rajauria[2]*, Erhard Schreck[2], Robert Smith[2], Qing Dai[2], David B. Bogy[1]

[1] Computer Mechanics Laboratory, University of California at Berkeley, Berkeley, CA 94720, USA

[2] Western Digital Corporation, Recording Sub System Staging and Research, San Jose, CA 95135, USA

*Correspondence: qlcheng@berkeley.edu (Q. Cheng), Sukumar.Rajauria@wdc.com (S. Rajauria)

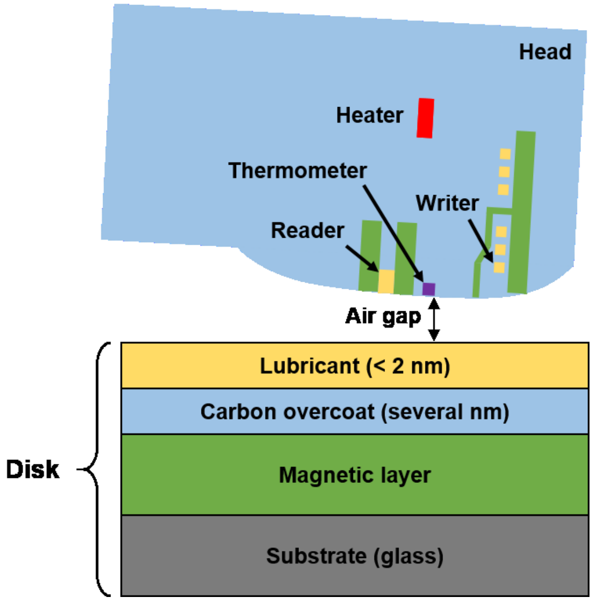

Fig. S1. A schematic of the head-disk interface. The head flies over the disk at a nanoscale air gap. The air gap is the physical spacing between the head surface and the disk surface. The initial air gap is ~10-15 nm when the heater is off. When the heater is turned on, it generates a thermal protrusion to gradually close the air gap until contact occurs. The disk has multiple layers such as lubricant, carbon overcoat, magnetic layer, and substrate.

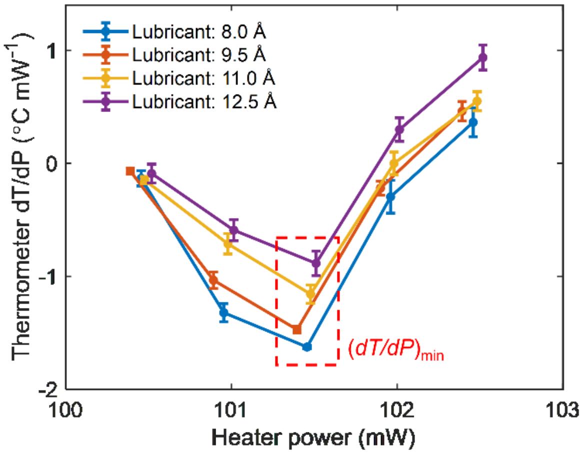

Fig. S2. The measured thermometer *dT/dP* versus the heater power at the small head-disk gap for lubricant thickness 8.0-12.5 Å. Contact occurs at 102.0 mW. The $(dT/dP)_{min}$ corresponds to the maximal thermal transport including air conduction, phonon heat conduction, contact heat conduction and frictional heating. The error bars are standard deviation of three repeated independent experiments.

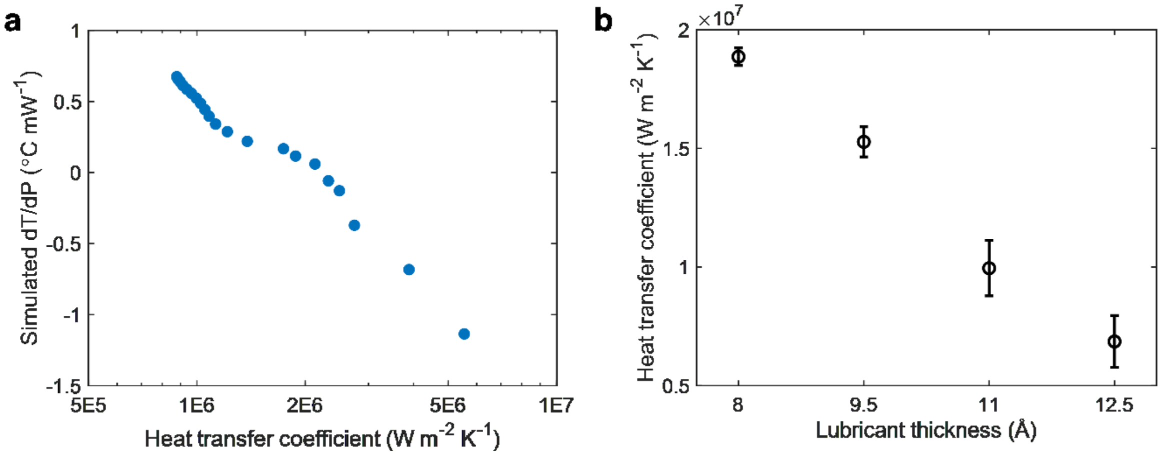

Fig. S3. Heat transfer coefficient (HTC) simulation results. **a** The simulation result of *dT/dP* versus HTC. **b** The maximal total HTCs for the four lubricant thicknesses. The HTC error bars correspond to the *dT/dP* error bars measured in Fig. S2.

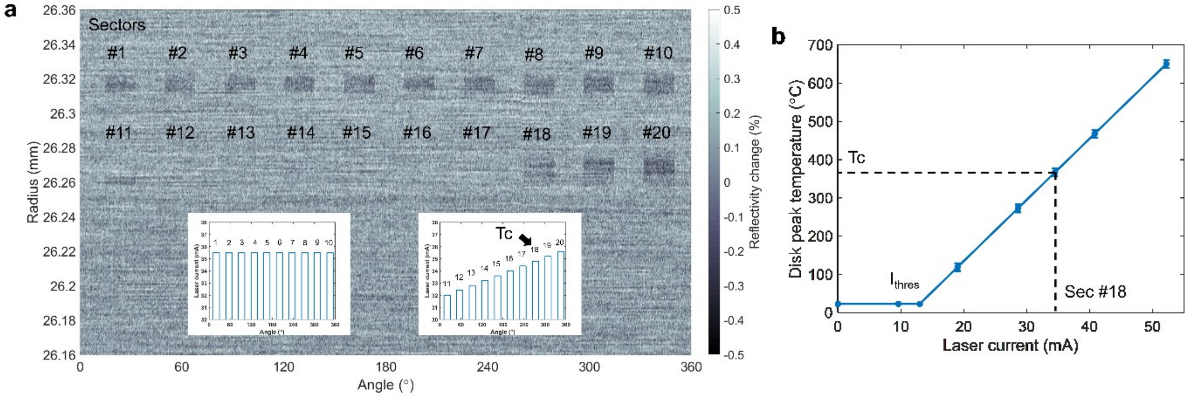

Fig. S4. Disk temperature calibration by HAMR writing. **a** The magnetic image of sectors under HAMR writing with different laser currents, taken by an optical surface analyzer Candela 5100. Inset: the laser currents for the sectors. The laser current in sector #18 corresponds to Curie temperature on the disk. **b** The calibrated disk peak temperature, which is assumed to be linear with the laser current. The error bars are the uncertainty in the disk temperature calibration, which is estimated to be below 10 °C from the laser current variation ~0.4 mA. The figure is reproduced from Ref[S1] with the permission from Elsevier.

Supplementary References

S1 Cheng, Q. & Bogy, D. B. Experimental study of smear formation and removal in heat-assisted magnetic recording. *Tribology International* **165**, 107258 (2022).
