# Supplementary material for: In-situ sub-angstrom characterization of laser-lubricant interaction in a thermo-tribological system
Source: Commun Eng. 2024 Oct 5;3:138. doi: 10.1038/s44172-024-00284-3 (PMC11457497; doi:10.1038/s44172-024-00284-3)
Supplement: Supplementary file 2 — Reporting Summary [file 44172_2024_284_MOESM2_ESM.pdf]

nature research

Corresponding author(s): Qilong Cheng, Sukumar Rajauria
COMMSENG-23-0507A

Lasing Reporting Summary

Nature Research wishes to improve the reproducibility of the work that we publish. This form is intended for publication with all accepted papers reporting claims of lasing and provides structure for consistency and transparency in reporting. Some list items might not apply to an individual manuscript, but all fields must be completed for clarity.

For further information on Nature Research policies, including our data availability policy, see Authors & Referees.

Experimental design

Please check: are the following details reported in the manuscript?

1. Threshold

Plots of device output power versus pump power over a wide range of values indicating a clear threshold

☒ Yes ☐ No — Fig. S4(b)

2. Linewidth narrowing

Plots of spectral power density for the emission at pump powers below, around, and above the lasing threshold, indicating a clear linewidth narrowing at threshold

☐ Yes ☒ No — Not relevant

Resolution of the spectrometer used to make spectral measurements

☐ Yes ☒ No — Not relevant

3. Coherent emission

Measurements of the coherence and/or polarization of the emission

☐ Yes ☒ No — Not relevant

4. Beam spatial profile

Image and/or measurement of the spatial shape and profile of the emission, showing a well-defined beam above threshold

☐ Yes ☒ No — Not relevant

5. Operating conditions

Description of the laser and pumping conditions
Continuous-wave, pulsed, temperature of operation

☐ Yes ☒ No — Not relevant

Threshold values provided as density values (e.g. W cm^{-2} or J cm^{-2}) taking into account the area of the device

☐ Yes ☒ No — Not relevant

6. Alternative explanations

Reasoning as to why alternative explanations have been ruled out as responsible for the emission characteristics
e.g. amplified spontaneous, directional scattering; modification of fluorescence spectrum by the cavity

☐ Yes ☒ No — Not relevant

7. Theoretical analysis

Theoretical analysis that ensures that the experimental values measured are realistic and reasonable
e.g. laser threshold, linewidth, cavity gain-loss, efficiency

☐ Yes ☒ No — Not relevant

8. Statistics

Number of devices fabricated and tested

☐ Yes ☒ No — Not relevant

Statistical analysis of the device performance and lifetime (time to failure)

☐ Yes ☒ No — Not relevant
